# Supplementary material for: Learning non-local molecular interactions via equivariant local representations and charge equilibration
Source: NPJ Comput Mater. 2025 Sep 16;11(1):287. doi: 10.1038/s41524-025-01790-4 (PMC12440812; doi:10.1038/s41524-025-01790-4)
Supplement: Supplementary file 1 — Supplementary Information [file 41524_2025_1790_MOESM1_ESM.pdf]

Supplementary Information

Learning Non-Local Molecular Interactions via Equivariant Local Representations and Charge Equilibration

Paul Fuchs[1], Michał Sanocki[1], Julija Zavadlav[1,2*]

[1]Multiscale Modeling of Fluid Materials, Department of Engineering Physics and Computation, TUM School of Engineering and Design, Technical University of Munich, Germany.
[2]Atomistic Modeling Center, Munich Data Science Institute, Technical University of Munich, Germany.

*Corresponding author(s). E-mail(s): julija.zavadlav@tum.de;

Supplementary Note 1

Predicted electronegativity, hardness, and covalent radii for the SPICE dataset

To evaluate the physically meaningfulness of the predicted electronegativity, hardness, and atomic radii during simulations of samples from the SPICE dataset, we compare the predictions against literature values in Figure 2.

Our findings indicate a correlation between the predicted hardness and atomic radii and their corresponding reference values, suggesting that the model effectively captures underlying physical trends for these properties. In contrast, the predicted electronegativity values exhibit a weaker correlation with classical electronegativity scales. These results align with the understanding that electronegativity is not an atom property that can be defined unambiguously or measured experimentally [1]. Moreover, the electronegativity is often not meaningful for atoms in isolation but within a specific chemical environment, which can differ within and between molecules. Electronegativities depend on, e.g., the valence state of atoms, which varies with the type of bonding [2]. Thus, chemical theory and our results support the approach of previous works [3–5] and this paper to model the highly environment-dependent electronenegativities through neural networks.

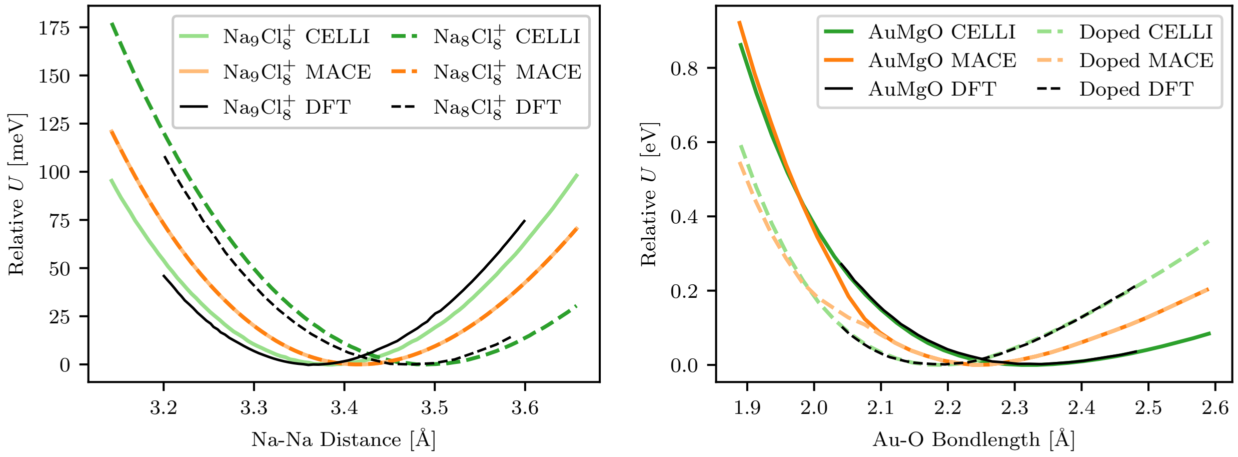

Supplementary Figure 1: Long-range interaction benchmarks. Evaluation of CELLI-enhanced MACE [6] on long-range benchmark systems [4]. **Left:** Relative energies for $Na_8Cl_8^+$ and $Na_9Cl_8^+$ clusters as a function of the Na–Na distance along a predefined path (indicated by arrow). CELLI-enhanced MACE reproduces DFT energy profiles and correctly identifies distinct minima for the two charge states, unlike baseline MACE. **Right:** Predicted bond energies for a gold dimer on an MgO(001) surface in the upright (non-wetting) geometry, with and without Al doping. CELLI-enhanced MACE matches DFT results for both cases, while baseline MACE fails to differentiate between doped and undoped substrates.

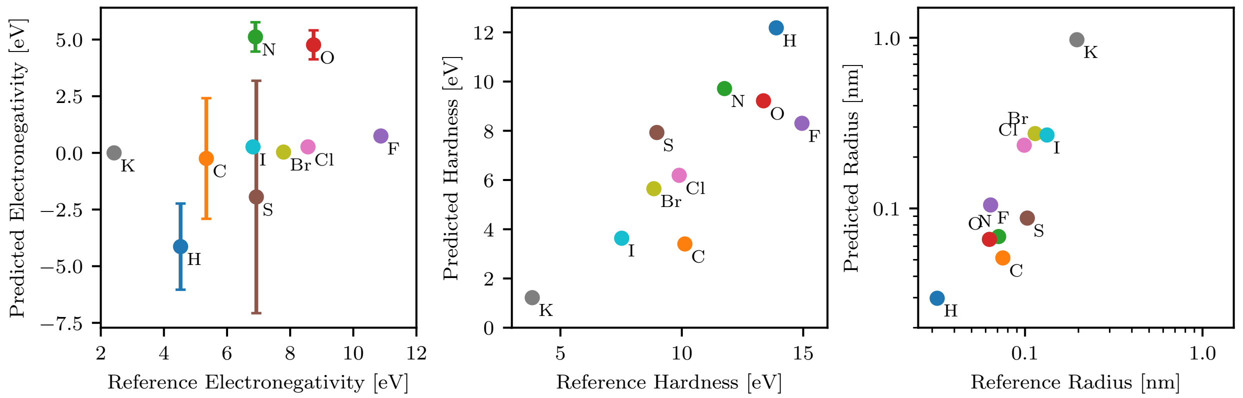

Supplementary Figure 2: Mean electronegativities with standard deviations predicted during simulations of systems from the *PubChem Sets, Amino Acid Ligands, Dipeptides, and DES370K* subsets of the SPICE dataset against calculated generalized Mulliken-Pauling electronegativities [7] (left), per-species hardness values against generalized Mulliken-Pauling hardness values [7] (middle), and per-species radii against experimentally determined single-bond covalent radii [8] (right).

Supplementary Table 1: Summary of accuracy and inference costs for Allegro [9], MACE [6], and DimeNet++ [10] on the OE62 dataset [11]. The *Small* (S) versions of CELLI and Allegro were used to compute the benchmarks and use fewer irreps, a lower rotational order for the spherical harmonics, and a smaller hidden size for the charge-embedding networks than the *Large* (L) version. The versions *S+* and *L+* of Allegro contain one additional Interaction Layer compared to the CELLI version. The times are reported per structure for a single sample per batch (s) and the optimal batch size with the highest throughput for the respective model (o) on 5000 samples up to 174 atoms. Energies given in meV, times in ms, and numbers of params in millions. The lowest errors are reported in bold. (*) Reported by Kosmala et al..

OE62	**Allegro**						**DimeNet++**	**MACE**	
	CELLI S	CELLI L	S	S+	L	L+		CELLI	
U MAE	55.3	55.1	63.4	60.0	61.1	61.8	**42.1** (53.8*)	48.0	48.1
U RMSE	116.7	114.3	123.7	114.5	120.9	116.6	108.4	**88.3**	90.1
Time (s)	3.3	4.3	2.6	3.7	3.6	5.1	7.7	2.8	**2.3**
Time (o)	2.9	4.3	2.6	3.7	3.6	5.1	6.9	1.6	**1.4**
# Params.	0.21	0.29	0.17	0.20	0.19	0.22	2.78	2.52	2.37

Supplementary Table 2: Model hyperparameters for the Carbon Chains (CH), Silver Clusters (Ag), NaCl Clusters (NaCl), Gold Dimers (AuMgO) [4], OE62 [11], and SPICE [13, 14] datasets. Coulomb cutoff *max* refers to a cutoff greater than the largest distance between any pair of atoms in the dataset. The number of Interaction Layers, respectively Message Passing (MP) layers, before and after CELLI are given as tuples. The dashed lines indicate that no combination of system and model architecture was trained.

	CH	Ag	NaCl	AuMgO	OE62	SPICE
**Allegro** [9]						
r_{cut} GNN (nm)	0.423	0.529	0.529	0.423	0.6	0.5
r_{cut} Coul (nm)	2.5	2.5	3.5	0.45^{a}	max	max
Irreps	$64 \times 1o$	$128 \times 1o$	$128 \times 1o$	$128 \times 1o$	$64 \times 1o$	$64 \times 1o$
					$+32 \times 2e$	$+16 \times 2e$
					$+16 \times 2o$	
					$(+16 \times 2o)^{\text{c}}$	
MLP dim	64	128	128	64	64	128
MLP layers	2	3	2	2	2	2
$\text{MLP}_{\mathcal{R}}$ dim	64	128	128	128	$64/128^{\text{c}}$	64
$\text{MLP}_{\mathcal{R}}$ layers	3	2	3	3	$3/2^{\text{c}}$	2
Interaction Layers	(2, 1)	(1, 1)	(2, 1)	(2, 1)	(2, 1)	(2, 1)
γ_U	0.1	1.0	0.1	0.01	$0.01/0.05^{\text{b}}$	0.0001
γ_F	0.001	0.001	0.0005	0.001		0.0001
γ_Q	50.0	10.0	10.0	200.0	0.1	0.1
**MACE** [6]						
r_{cut} GNN (nm)	0.423	—	0.529	0.423	0.6	—
r_{cut} Coul (nm)	2.5	—	3.5	0.45^{*}	max	—
Irreps	$64 \times 0e$	—	$64 \times 0e$	$64 \times 0e$	$128 \times 0e$	—
	$+32 \times 1o$		$+32 \times 1o$	$+32 \times 1o$	$+32 \times 1o$	
$\text{MLP}_{\mathcal{R}}$ dim	64	—	64	64	128	—
$\text{MLP}_{\mathcal{R}}$ layers	$3/2^{\text{d}}$	—	2	2	2	—
MP Layers	(1, 1)	—	(1, 1)	(1, 1)	(1, 1)	—
	/(3, 3)		/(3, 3)	/(3, 3)		
γ_U	0.1	—	0.1	0.01	0.05	—
γ_F	0.001	—	0.0005	0.001		—-
γ_Q	50.0	—	10.0	200.0	100.0	—

[a]Combined with long-range electrostatic treatment via the SPME method.

[b]Models combined with CELLI.

[c]Large Allegro models.

[d]MACE with 6 message-passing layers.

Supplementary Table 3: Root mean square errors (RMSE) in units of meV/atom, meV/Å, and me, for CELLI with per-species hardness (J^Z) and environment-dependent hardness ($J^{\mathcal{R}}$) applied to the strictly local Allegro model and MACE model. The numbers of message-passing steps, if applicable, are given in brackets behind the model name. The CELLI variants embed the charge environment (Environment) or only the charge of the central atom (Local). The lowest errors for the MACE and Allegro variants are reported in bold.

	**Allegro** (–)				**MACE** (2)	
	Environment		Local		Environment	Local
	J^Z	$J^{\mathcal{R}}$	J^Z	$J^{\mathcal{R}}$	J^Z	J^Z
**Carbon Chains**						
Energy U	0.599	0.609	**0.575**	0.580	**0.398**	0.419
Force F	31.00	32.31	**29.87**	30.13	**21.45**	23.65
Charge Q	4.003	3.451	3.821	**3.192**	3.458	**3.453**
**Silver Clusters**						
Energy U	0.80	0.81	**0.787**	0.824	–	–
Force F	20.33	**20.11**	20.47	20.50	–	–
Charge Q	6.360	**1.727**	6.606	2.228	–	–
**NaCl Clusters**						
Energy U	0.127	**0.114**	0.158	0.148	**0.097**	0.114
Force F	6.444	**5.15**	8.58	7.56	**3.54**	5.02
Charge Q	15.72	9.15	16.350	**6.165**	**15.520**	15.571
**Gold Dimers**						
Energy U	**0.077**	**0.077**	0.094	0.106	**0.069**	0.072
Force F	12.04	**12.01**	17.46	17.95	**7.95**	8.56
Charge Q	5.510	**4.542**	5.679	4.771	**5.171**	5.279

Supplementary Table 4: Root mean square errors (RMSE) in units of meV/atom, meV/Å, and me, for CELLI placed at different locations in the strictly local Allegro model and MACE model. The placement of CELLI is given through a tuple with the number of Interaction Layers, respectively message-passing layers, before and after CELLI. The lowest errors for the MACE and Allegro variants are reported in bold.

	**Allegro**				**MACE**		
	(0, 3)	(1, 2)	(2, 1)	(3, 0)	(0, 2)	(1, 1)	(2, 0)
**NaCl Clusters**							
Energy U	0.167	**0.108**	0.114	0.126	0.362	**0.097**	0.114
Force F	9.99	**4.76**	5.15	7.06	14.42	**3.54**	4.32
Charge Q	20.349	**5.706**	9.150	6.148	48.058	**15.520**	15.834
**Gold Dimers**							
Energy U	0.079	0.086	**0.077**	0.086	0.074	0.069	**0.066**
Force F	14.13	**11.05**	12.01	13.91	8.07	7.95	**6.12**
Charge Q	5.73	**3.820**	4.542	4.019	10.655	5.171	**4.123**

References

[1] Politzer, P., Murray, J.S.: Electronegativity—a perspective. Journal of Molecular Modeling **24**(8), 214 (2018) https://doi.org/10.1007/s00894-018-3740-6

[2] Hinze, Jurgen., Jaffe, H.H.: Electronegativity. I. Orbital Electronegativity of Neutral Atoms. Journal of the American Chemical Society **84**(4), 540–546 (1962) https://doi.org/10.1021/ja00863a008

[3] Ghasemi, S.A., Hofstetter, A., Saha, S., Goedecker, S.: Interatomic potentials for ionic systems with density functional accuracy based on charge densities obtained by a neural network. Phys. Rev. B **92**, 045131 (2015) https://doi.org/10.1103/PhysRevB.92.045131

[4] Ko, T.W., Finkler, J.A., Goedecker, S., Behler, J.: A fourth-generation high-dimensional neural network potential with accurate electrostatics including non-local charge transfer. Nature Communications **12**(1), 398 (2021) https://doi.org/10.1038/s41467-020-20427-2

[5] Shaidu, Y., Pellegrini, F., Küçükbenli, E., Lot, R., Gironcoli, S.: Incorporating long-range electrostatics in neural network potentials via variational charge equilibration from shortsighted ingredients. npj Computational Materials **10**(1), 47 (2024) https://doi.org/10.1038/s41524-024-01225-6

[6] Batatia, I., Kovács, D.P., Simm, G.N.C., Ortner, C., Csányi, G.: MACE: Higher Order Equivariant Message Passing Neural Networks for Fast and Accurate Force Fields (2023). https://arxiv.org/abs/2206.07697

[7] Rappe, A.K., Goddard III, W.A.: Charge equilibration for molecular dynamics simulations. The Journal of Physical Chemistry **95**(8), 3358–3363 (1991) https://doi.org/10.1021/j100161a070

[8] Pyykkö, P., Atsumi, M.: Molecular Single-Bond Covalent Radii for Elements 1–118. Chemistry – A European Journal **15**(1), 186–197 (2009) https://doi.org/10.1002/chem.200800987

[9] Musaelian, A., Batzner, S., Johansson, A., Sun, L., Owen, C.J., Kornbluth, M., Kozinsky, B.: Learning local equivariant representations for large-scale atomistic dynamics. Nature Communications **14**(1), 579 (2023) https://doi.org/10.1038/s41467-023-36329-y

[10] Gasteiger, J., Giri, S., Margraf, J.T., Günnemann, S.: Fast and Uncertainty-Aware Directional Message Passing for Non-Equilibrium Molecules. arXiv (2022). https://doi.org/10.48550/arXiv.2011.14115

[11] Stuke, A., Kunkel, C., Golze, D., Todorović, M., Margraf, J.T., Reuter, K., Rinke, P., Oberhofer, H.: Atomic structures and orbital energies of 61,489 crystal-forming organic molecules. Scientific Data **7**(1), 58 (2020) https://doi.org/10.1038/s41597-020-0385-y

[12] Kosmala, A., Gasteiger, J., Gao, N., Günnemann, S.: Ewald-based long-range message passing for molecular graphs. In: Krause, A., Brunskill, E., Cho, K., Engelhardt, B., Sabato, S., Scarlett, J. (eds.) Proceedings of the 40th International Conference on Machine Learning. Proceedings of Machine Learning Research, vol. 202, pp. 17544–17563. PMLR, Honolulu, Hawaii, USA (2023). https://proceedings.mlr.press/v202/kosmala23a.html

[13] Eastman, P., Behara, P.K., Dotson, D.L., Galvelis, R., Herr, J.E., Horton, J.T., Mao, Y., Chodera, J.D., Pritchard, B.P., Wang, Y., De Fabritiis, G., Markland, T.E.: SPICE, A Dataset of Drug-like Molecules and Peptides for Training Machine Learning Potentials. Scientific Data **10**(1), 11 (2023) https://doi.org/10.1038/s41597-022-01882-6

[14] Eastman, P., Behara, P.K., Dotson, D., Galvelis, R., Herr, J., Horton, J., Mao, Y., Chodera, J., Pritchard, B., Wang, Y., De Fabritiis, G., Markland, T.: SPICE 2.0.1. https://doi.org/10.5281/zenodo.10975225 . https://doi.org/10.5281/zenodo.10975225
